# Global prevalence of fluoroquinolone resistance in *Escherichia coli* causing urinary tract infections: a systematic review and meta-analysis

**DOI:** 10.3389/fcimb.2026.1831616

**Published:** 2026-05-20

**Authors:** Hua Zhou, Tianrui Xue, Jingxin Xue

**Affiliations:** 1 Department of Hospital-Acquired Infection Control, Jinan Third People’s Hospital, Jinan Third People’s Hospital Affiliated with Jining Medical College, Jinan, Shandong, China; 2 School of Clinical Medicine, Shandong Second Medical University, Weifang, Shandong, China; 3 Department of Urology Surgery, Jinan Third People’s Hospital, Jinan Third People’s Hospital Affiliated with Jining Medical College, Jinan, Shandong, China

**Keywords:** drug resistance, *Escherichia coli*, fluoroquinolone resistance, meta-analysis, urinary tract infections

## Abstract

**Background:**

Fluoroquinolone resistance in *Escherichia coli* isolated from urinary tract infections (UTIs) is an increasing global concern, with marked variation across regions and specific agents. Estimating resistance prevalence is essential to guide empirical therapy and strengthen antimicrobial stewardship programs.

**Methods:**

A systematic review and meta-analysis was conducted in accordance with PRISMA guidelines, including English-language observational studies published through December 2025. PubMed, Embase, and Web of Science were searched for reports on fluoroquinolone-resistant *E. coli* in UTIs. Study quality was evaluated using the modified Newcastle-Ottawa Scale. Pooled prevalence estimates were calculated using a random-effects model, and heterogeneity was assessed with the I² statistic. Subgroup analyses were performed by fluoroquinolone agent. Robustness was examined through sensitivity analyses, while meta-regression explored associations between sample size and resistance prevalence. Publication bias and influential studies were evaluated using DOI and Baujat plots.

**Results:**

Among 9,033 identified records, 36 studies met the inclusion criteria. The overall pooled prevalence of fluoroquinolone resistance in *E. coli* was 31.09% (95% CI, 24.89%–38.05%). Resistance estimates were 30.32% (95% CI, 22.60%–39.34%) for ciprofloxacin, 27.57% (95% CI, 9.01%–59.40%) for levofloxacin, and 68.75% (95% CI, 55.94%–79.76%) for pefloxacin. Sensitivity analyses demonstrated stable pooled estimates ranging from 30% to 32%. Meta-regression suggested an inverse association between study sample size and reported resistance rates. One study contributed modestly to heterogeneity.

**Conclusion:**

This analysis demonstrates substantial and geographically heterogeneous fluoroquinolone resistance in *E. coli* causing UTIs, with particularly high rates reported in China, Iran, and Bangladesh. While ciprofloxacin resistance was considerable, pefloxacin exhibited the highest prevalence. These findings reinforce the importance of enhanced stewardship initiatives, improved resistance surveillance, harmonized reporting standards, and further investigation into resistance mechanisms and data gaps in underrepresented regions.

## Introduction

Urinary tract infections (UTIs) affect millions of individuals worldwide, with women being the most impacted group (Baimakhanova et al., 2025). These infections range from mild cases in healthy individuals to severe cases in people with underlying health conditions or structural abnormalities in the urinary tract (Kamei and Fujimura, 2023; Baimakhanova et al., 2025). Without proper treatment, UTIs lead to serious complications such as kidney infections, long-term damage, or life-threatening conditions like sepsis (Ashraf et al., 2020; Wagenlehner et al., 2020; Zhou et al., 2023). The widespread prevalence of UTIs highlights the need for effective treatments that reduce health risks and improve the financial strain on healthcare systems (Shafrin et al., 2022).

*Escherichia coli* causes the majority of UTIs, accounting for most community-acquired and hospital-related infections (Milano et al., 2022). The bacterium infects the urinary tract by producing virulence factors, such as biofilms that protect it from antibiotics and immune responses, and molecules that allow it to adhere to urinary tissues (Govindarajan and Kandaswamy, 2022; Timm et al., 2024). Antibiotics have traditionally treated *E. coli* infections effectively, but rising antimicrobial resistance has made treatment more challenging and increased treatment failures (Ahmed et al., 2024). These challenges emphasize the need for targeted approaches to manage resistant infections (Maillard et al., 2020; Zhao et al., 2026).

Fluoroquinolones, once a highly effective treatment for UTIs due to their broad-spectrum antibacterial properties and ability to concentrate in urinary tissues, are now losing reliability as resistance rates increase (Bhatt and Chatterjee, 2022; Wang et al., 2025). Overprescribing fluoroquinolones for mild cases, their unregulated availability, and incomplete treatment courses have accelerated resistance development (Damlin, 2020; Muteeb et al., 2023). Bacteria like *E. coli* have adapted by developing genetic mutations and transferring resistance traits via plasmids, making infections harder to treat (Urban-Chmiel et al., 2022; Nasrollahian et al., 2024). This systematic review and meta-analysis quantify the prevalence of fluoroquinolone resistance in UTIs. By synthesizing data from existing studies, this analysis offers valuable insights into current resistance rates, guiding better antibiotic use and informing strategies to manage UTIs effectively.

## Method

This meta-analysis was conducted following PRISMA guidelines (**Supplementary Table 1**).

### Eligibility criteria

The studies included data on the prevalence of fluoroquinolone resistance in *Escherichia coli* isolates from patients with UTIs. Eligible studies were observational, including cross-sectional and cohort designs, published in English within the past 15 years, up to December 2025.The research question for this systematic review and meta-analysis was structured using the PICOS framework to enhance clarity and methodological rigor. The population (P) included patients with urinary tract infections caused by Escherichia coli. The intervention/exposure (I) was the use of fluoroquinolone antibiotics, including ciprofloxacin, levofloxacin, and norfloxacin. The comparator (C) was defined as comparisons across different fluoroquinolone agents, study settings (e.g., community vs hospital), and study populations where applicable. The outcome (O) was the prevalence of fluoroquinolone resistance. The study design (S) included observational studies reporting resistance data. This structured approach ensured consistency in study selection, data extraction, and analysis. The studies specifically focused on *E. coli* and its resistance rates. Excluded studies consisted of letters to the editor, commentaries, qualitative studies, case series, case reports, reviews, discussion papers, clinical trials, abstracts with insufficient data, and articles lacking full-text availability or falling outside the specified timeframe and language criteria (**Supplementary Table 2****).**

### Search strategy

A systematic search was performed across PubMed, Embase, and Web of Science databases to gather studies relevant to fluoroquinolone resistance in *Escherichia coli*. The search timeframe included all publications available up to December 2025. Keywords applied in the search included: “antibiotic resistance,” “antimicrobial resistance,” “bacterial resistance,” “drug resistance, microbial”, “drug resistance, bacterial”, “fluoroquinolone resistance,” “ciprofloxacin resistance,” “levofloxacin resistance,” and “moxifloxacin resistance.” These were combined with terms such as “urinary tract infection,” “bacteriuria,” “pyuria,” as well as their corresponding MeSH terms, alongside “Escherichia coli” and “Enteroaggregative Escherichia coli.” To ensure transparency and reproducibility, the full database-specific search strategies are provided in the Supplementary Materials (**Supplementary Table 3**). The search was restricted to English-language publications due to resource constraints and to ensure accuracy in data extraction and interpretation. This restriction may introduce language bias and has been acknowledged as a limitation. In addition to electronic database searches, a systematic manual search of the reference lists of included studies and relevant reviews was conducted to identify any additional eligible articles that may have been missed during the initial search process.

### Screening and data extraction

All studies were screened and managed using Nested Knowledge software, automatically removing duplicates. Two reviewers independently assessed titles and abstracts for relevance, followed by a review of full-text articles for eligibility. Discrepancies were resolved through discussion or consultation with a third reviewer if necessary. Data extraction was performed using the same software, focusing on study characteristics (author, year, location, design, sample size), population details (age, study duration, healthcare setting), and fluoroquinolone resistance rates. Two reviewers independently extracted data, resolving inconsistencies through discussion.

For studies reporting resistance to multiple fluoroquinolone agents, data were extracted separately for each antibiotic (e.g., ciprofloxacin, levofloxacin, norfloxacin), and no aggregation across antibiotics was performed. Each study contributed one effect estimate per antibiotic to the corresponding meta-analysis. Although multiple outcomes from the same study may introduce within-study correlation, each antibiotic was analyzed independently, minimizing the impact of such correlations on pooled estimates.

### Quality assessment

Study quality was assessed using a modified version of the Newcastle–Ottawa Scale (NOS), adapted for prevalence studies of antimicrobial resistance. The modification included domains such as Definition of UTIs and Ascertainment of Events to ensure accurate case identification and reliable measurement of resistance outcomes. Each study was evaluated based on predefined criteria, and scores were assigned accordingly. Detailed scoring criteria for each domain are provided in the Supplementary Materials (**Supplementary Table 4**) to ensure transparency and reproducibility.

The modified NOS assessed study quality across four domains: representativeness, adequate sample size, definition of UTIs, and ascertainment of events, with a maximum total score of 6 points. Studies scoring 0–2 points were considered low quality, 3–4 points moderate quality, and 5–6 points high quality. Two reviewers independently assessed all studies, and any disagreements were resolved through discussion.

### Statistical analysis

All analyses were performed using R software (Schwarzer, 2022). A random-effects model accounted for heterogeneity (Zhai and Guyatt, 2024), assessed with the I² statistic, with values above 50% indicating substantial heterogeneity (Mheissen et al., 2024). To account for the non-normal distribution of proportions and to stabilize the variance, prevalence data were pooled using a random-effects model with Freeman-Tukey double arcsine transformation. This approach is particularly robust for meta-analyses where proportions may approach the boundaries of 0 or 1, ensuring that studies with extremely low or high resistance rates are not assigned inappropriate weights. Following the calculation of the pooled effect size, results were back-transformed to proportions and expressed as percentages with 95% confidence intervals (CIs). This approach ensured robust estimation of resistance prevalence across studies. To address potential within-study dependence arising from reports of multiple fluoroquinolone agents within a single study, we performed independent meta-analyses for each antibiotic. This approach ensures that a single study never contributes more than one effect size to a specific pooled estimate, thereby maintaining the independence of the unit of analysis. While within-study correlation exists (i.e., isolates resistant to ciprofloxacin are likely resistant to levofloxacin), analyzing them in separate subgroups prevents this correlation from biasing the point estimates or artificially narrowing the confidence intervals of the drug-specific findings.

Subgroup analyses examined variations by fluoroquinolone class. DOI plots assessed publication bias (Shamim, 2023), sensitivity analysis tested result robustness (Meng et al., 2024), and meta-regression and Baujat plots identified influential studies All statistical analyses were conducted using R software (R Foundation for Statistical Computing, Vienna, Austria). Forest plots, Doi plots, and Baujat plots were generated using relevant functions within these packages. A p-value of <0.05 was considered statistically significant.

## Results

**Figure 1:**
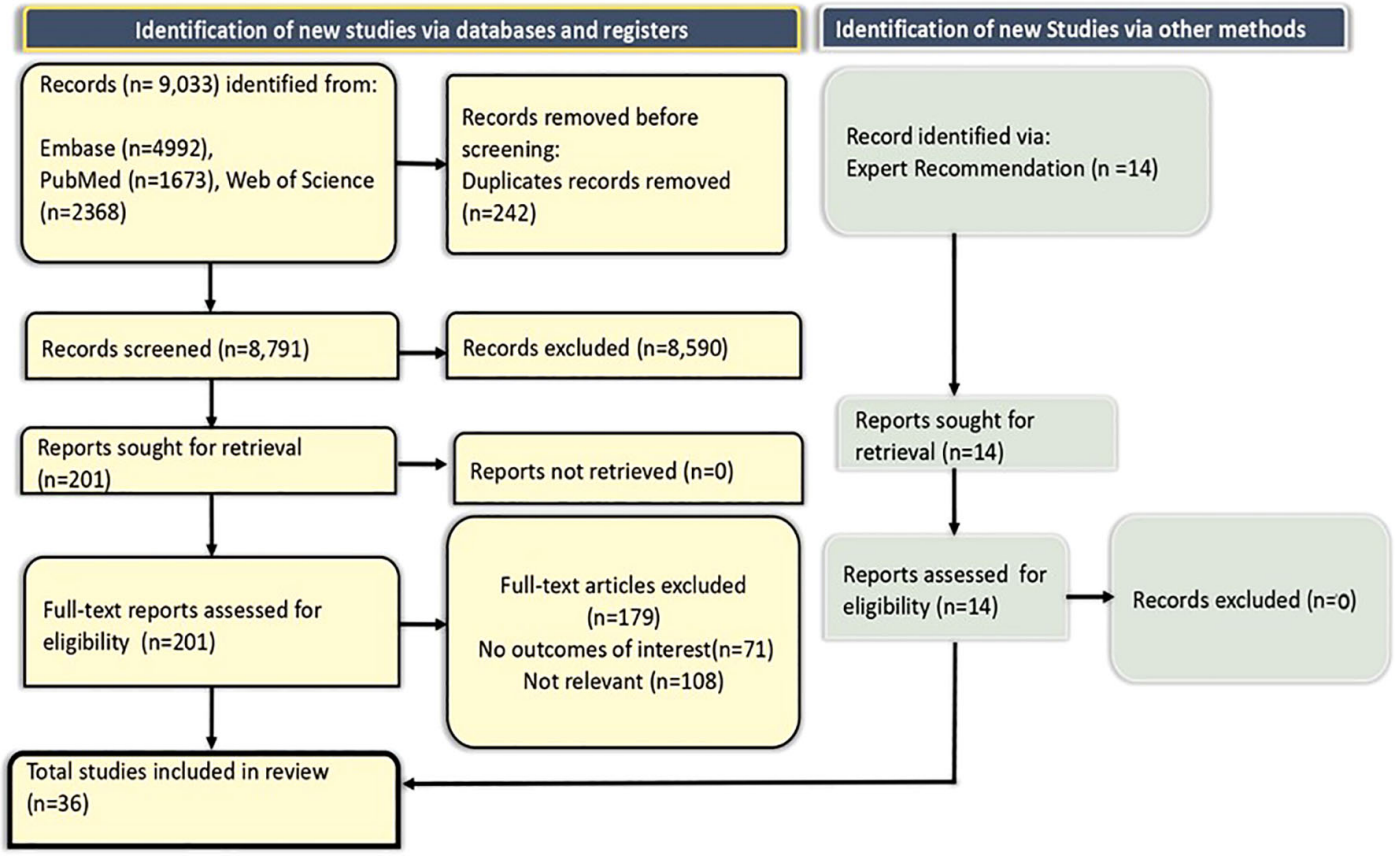
PRISMA flow diagram for the study selection process.

A total of 9,033 records were identified from Embase (n=4,992), PubMed (n=1,673), and Web of Science (n=2,368). After removing 242 duplicates, 8,791 records were screened, with 8,590 excluded. Full-text assessment was conducted for 201 reports, of which 179 were excluded (71 with no outcomes of interest and 108 not relevant). Fourteen additional records were identified through expert recommendations. Ultimately, 36 studies were included in the final review (**Figure 1**).

### Basic characteristics of the study

A total of 36 studies were analyzed, with contributions from North America (USA: 3 studies (Faine et al., 2022) (Fleming et al., 2014; Fromer et al., 2024), Canada: 1 study (Soucy et al., 2020), Mexico: 1 study (López-Banda et al., 2014); Europe (France: 3 studies (Etienne et al., 2014; Leforestier et al., 2020; Biguenet et al., 2023), Germany: 2 studies (Naber et al., 2023) (Kresken et al., 2014), and 1 study each from the UK (Findlay et al., 2020), Spain (Loras et al., 2020), Poland (Jurałowicz et al., 2020), and Romania (Cristea et al., 2019); Asia (China: 3 studies (Li et al., 2022) (Wang et al., 2014; Wang et al., 2021), Turkey: 4 studies (Can et al., 2015; Demirci et al., 2019; Akgoz et al., 2020; Demir and Kazanasmaz, 2020), Iran: 3 studies (Asadi et al., 2014; Sedighi et al., 2015; Afsharikhah et al., 2023), India: 2 studies (Harwalkar et al., 2014; Niranjan and Malini, 2014), Bangladesh: 2 studies (Rahman et al., 2014; Islam et al., 2022), and 1 study each from Nepal (Kushwaha et al., 2021) and Thailand (Themphachana et al., 2014); the Middle East (Saudi Arabia: 1 study (Bazaid et al., 2021), Egypt: 1 study (Kotb et al., 2019), Israel: 1 study (Brosh-Nissimov et al., 2019); and South America (Brazil: 2 studies (Rodrigues et al., 2016; de Souza da-Silva et al., 2020), Venezuela: 1 study (Guzmán et al., 2019). Patient ages ranged from under 18 to over 65 years, with sample sizes ranging from 29 to 113,361 *Escherichia coli* isolates. Fluoroquinolone resistance rates ranged from 9% to 68.75%, varying by region and antibiotic type. Study designs included cross-sectional, retrospective, and prospective observational studies, highlighting resistance patterns across diverse populations (**Table 1**).

**Table 1:** Basic characteristics included in the study.

Author	Country	Study design	Setting	Study duration	Mean age (year)	E. Coli isolates from UTIs patients (N)	Fluoroquinolone resistance (n)
Afsharikhah_2023 (Afsharikhah et al., 2023)	Iran	Cross-sectional study	Community	Spring season of 2021	NA	106	Ciprofloxacin = 58
Akgoz_2020 (Akgoz et al., 2020)	Turkey	Prospective observational study	Community	2015 and 2016	58	258	Fluoroquinolones = 97
Aminul islamid_2022 (Islam et al., 2022)	Bangladesh	Cross-sectional study	Community	September 2016 to November 2018	>18	2655	Fluoroquinolones = 1,831
Asadi_2014 (Asadi et al., 2014)	Iran	Cross-sectional study	Hospitalized	2010 and 2011	33	60	Ciprofloxacin = 13
Bazaid_2021 (Bazaid et al., 2021)	Saudi Arabia	Retrospective cohort study	Hospitals	January 2015 to December 2019	Adults 13 -65, Children <13	156	Ciprofloxacin = 26
Biguenet_2023 (Biguenet et al., 2023)	France	Retrospective study	Community setting,	2016 -2017	75	11361	Ciprofloxacin = 1533
Brosh-nissimov_2019 (Brosh-Nissimov et al., 2019)	Israel	Cross-sectional study	Community	2014–16	Median 20.2 IQR [19.6–21]	1004	Ciprofloxacin = 111
Can_2015 (Can et al., 2015)	Turkey	Cohort study	Community	2011	50	294	Ciprofloxacin = 114
Corina cristea_2019 (Cristea et al., 2019)	Romania	Cross-sectional study	Community	One month in 2018	16--90	787	Ciprofloxacin =118 levofloxacin = 117
Demir_2020 (Demir and Kazanasmaz, 2020)	Turkey	Retrospectively study	Community and hospital	May 2015 to May 2017	Na	496	Ciprofloxacin = 77 levofloxacin = 5
Demirci_2019 (Demirci et al., 2019)	Turkey	Cross-sectional study	Community and hospital	April and august 2018	37	101	Ciprofloxacin = 26
Etienne_2014 (Etienne et al., 2014)	France	Prospective observational study	Community	2009 to 2011	18 and 65	157	Ofloxacin = 97 levofloxacin = 97
Faine_2022 (Faine et al., 2022)	USA	Retrospective study	Hospital	2018 to 2020	Median 62.9 IQR 41– 77.6	1471	Ciprofloxacin = 269 levofloxacin = 157 ciprofloxacin or levofloxacin = 313
Findlay_2019 (Findlay et al., 2020)	UK	Cross-sectional study.	Community	Sept 2017 and august 2018	63	225	Ciprofloxacin =128
Fleming_2014 (Fleming et al., 2014)	USA	Retrospective cohort study.	Community	September 2017 - august 2018	< 18	156	21
G. Naber_2023 (Naber et al., 2023)	Germany	Retrospective study	Community	January 2017 to December 2019	≥ 12	345	Ciprofloxacin = 18
Harwalkar_2014 (Harwalkar et al., 2014)	India	Cross-sectional study	Community and hospital	December 2010 to February 2013	35	193	Ciprofloxacin = 97
Jurałowicz_2020 (Jurałowicz et al., 2020)	Poland	Retrospective study	Community	2016 to 2018	Median IQR 65 (59–77)	387	Ciprofloxacin = 154
Kresken_2014 (Kresken et al., 2014)	Germany	Cross-sectional study	Community	October to December 2010	Median 59	499	98
Kushwaha_2021 (Kushwaha et al., 2021)	Nepal	Cross-sectional study	Hospital setting	October 2018 to February 2019	21-30	71	Norfloxacin = 37 ciprofloxacin = 23
L. Fromer_2024 (Fromer et al., 2024)	USA	Retrospective study	Community	October 2015–February 2020	≥12	Recurrent UTIs = 12,234 and non-recurrent UTIs = 68,033	Fluoroquinolones = 1,737 (recurrent) and 5,850 (nonrecurrent)
Leforestier_2020 (Leforestier et al., 2020)	France	Prospective study	Community	March to August 2018, (district #2), April to August 2019 (district #1)	Median 39 IQR (25 –70)	166	Fluoroquinolones = 19
Li_2022 (Li et al., 2022)	China	Retrospective study	A hospital setting,	3 years	73	519	Ciprofloxacin = 339 levofloxacin = 318
López-banda_2014 (López-Banda et al., 2014)	Mexico	Retrospective study	Hospital setting	2008 to 2010	39	Ciprofloxacin = 106 Gatifloxacin = 64 levofloxacin = 108 Moxifloxacin = 19	Ciprofloxacin = 66 Gatifloxacin = 40 levofloxacin = 65 Moxifloxacin = 10
Loras_2020 (Loras et al., 2020)	Spain	Retrospective study	Community and hospital settings	2017	NA	39	Ciprofloxacin = 31
Nabil kotb_2019 (Kotb et al., 2019)	Egypt	Cross-sectional study	Community and hospital	July 2016 to march 2017	> 18	281	Ciprofloxacin = 54 norfloxacin = 54 ofloxacin = 54
Niranjan_2014 (Niranjan and Malini, 2014)	India	Cross-sectional study	Hospital setting	August 2011 to July 2012	NA	119	Norfloxacin = 88
Rahman_2014 (Rahman et al., 2014)	Bangladesh	Cross-sectional study	Community-based study,	February 2011 to December 2011	19	29	Levofloxacin =20 Ciprofloxacin =19
Rodrigues_2016 (Rodrigues et al., 2016)	Brazil	Cross-sectional study	Hospital setting	January 2010 to December 2015	NA	1165	
Sedighi_2015 (Sedighi et al., 2015)	Iran	Cross-sectional study	Hospital setting,	October 2010 to October 2011	NA	120	Ciprofloxacin = 18 norfloxacin = 18 ofloxacin =17
Soucy_2020 (Soucy et al., 2020)	Canada	Cross-sectional study	Hospital	April 2010 to December 2014	15–65	11333	Ciprofloxacin = 2,085
Themphachana_2014 (Themphachana et al., 2014)	Thailand	Cross-sectional study	Hospital.	Na	>15	113	Norfloxacin = 65
Wang_2021 (Wang et al., 2021)	China	Retrospective study	Hospital	April to November 2008	NA	64	Levofloxacin = 22 norfloxacin = 18 pefloxacin =44 ciprofloxacin =19
Wang_2014 (Wang et al., 2014)	China	Cross sectional study	Community setting	NA	Median 64 IQR [53-77]	129	Ciprofloxacin = 91
Guzmán_2014 (Guzmán et al., 2019)	Venezuela	Retrospective study	Community setting	January 1^st^ - June 30^th^, 2014	12 -70	103	Ciprofloxacin = 30
da-Silva_2020 (de Souza da-Silva et al., 2020)	Brazil	Cross-sectional study	Community setting	November 2015	18 -59	499	Ciprofloxacin = 98 norfloxacin = 99

NA, Not applicable

IQR, Interquartile range

### Study quality assessment

Study quality was assessed using a modified NOS, and the results are summarized in **Supplementary Table 4**. Overall, the majority of included studies were of moderate to high quality, with total scores ranging from 4 to 5 out of a maximum of 6 points. Most studies demonstrated good representativeness and adequate outcome ascertainment, reflecting reliable identification of *Escherichia coli* isolates and resistance outcomes. However, variability was observed in certain domains, particularly sample size adequacy and case definition, where some studies did not fully meet the predefined criteria.

Overall, no studies were classified as low quality, indicating a generally acceptable methodological standard across the included literature. Given the relatively limited variability in quality scores and the predominance of moderate-to-high-quality studies, further subgroup or sensitivity analyses based on study quality were not considered feasible. Nonetheless, potential variations in study design and reporting may have contributed to the observed heterogeneity and should be considered when interpreting the pooled estimates.

### Meta-analysis

#### Prevalence of fluoroquinolone resistance in *E. coli* UTIs

**Figure 2:**
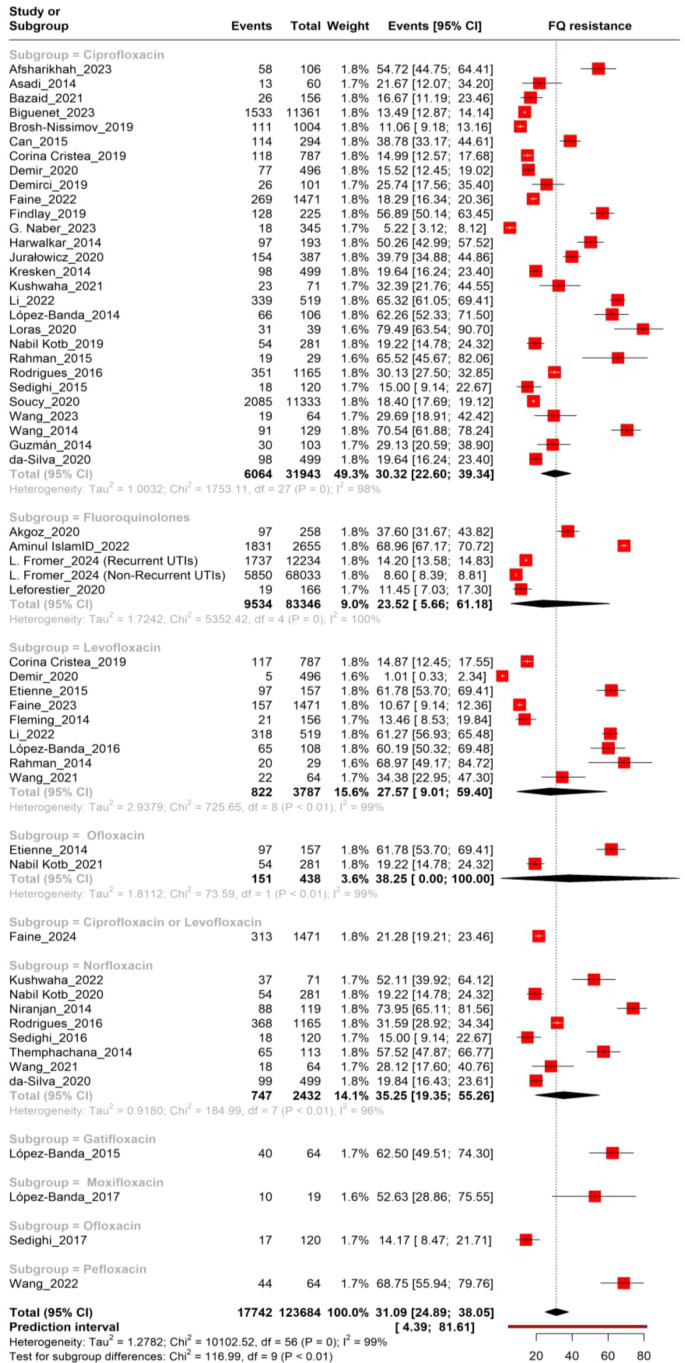
Forest plot depicting the prevalence of fluoroquinolone resistance in UTIs.

The meta-analysis showed a pooled prevalence of fluoroquinolone resistance in *Escherichia coli* isolates at 31.09% (95% CI: 24.89%–38.05%). Subgroup analysis revealed resistance rates of 30.32% (95% CI: 22.60%–39.34%) for ciprofloxacin, 27.57% (95% CI: 9.01%–59.40%) for levofloxacin, 38.25% (95% CI: 0.00%–100.00%) for ofloxacin, 35.25% (95% CI: 19.35%–55.26%) for norfloxacin, 62.50% (95% CI: 49.51%–74.30%) for gatifloxacin, 52.63% (95% CI: 28.86%–75.55%) for moxifloxacin, and 68.75% (95% CI: 55.94%–79.76%) for pefloxacin. Ciprofloxacin had the most stable data due to its larger sample size, while other subgroups showed wider confidence intervals due to fewer studies. High heterogeneity (I² = 99%) indicated substantial variability across studies, highlighting significant resistance levels and variations among fluoroquinolones (**Figure 2**).

### Sensitivity analysis

**Figure 3:**
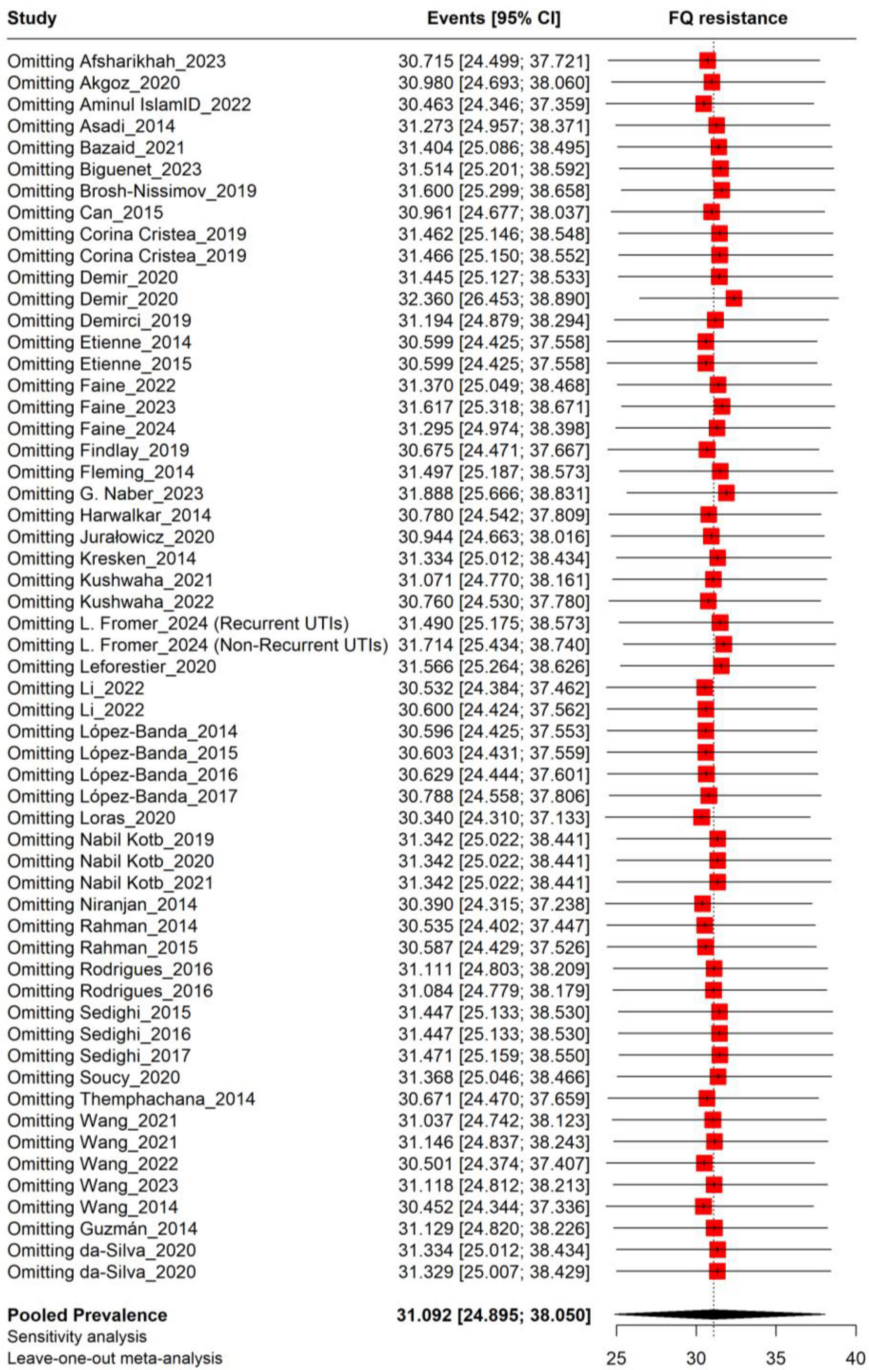
Sensitivity analysis plot for the prevalence of fluoroquinolone resistance in UTIs.

The sensitivity analysis using a leave-one-out meta-analysis estimated a pooled prevalence of fluoroquinolone resistance in *Escherichia coli* isolates from UTIs at 31.09% (95% CI: 24.90%–38.05%). Excluding individual studies had minimal effect, with prevalence estimates consistently ranging from 30% to 32%, showing no significant influence from any single study (**Figure 3**).

### Publication bias

**Figure 4:**
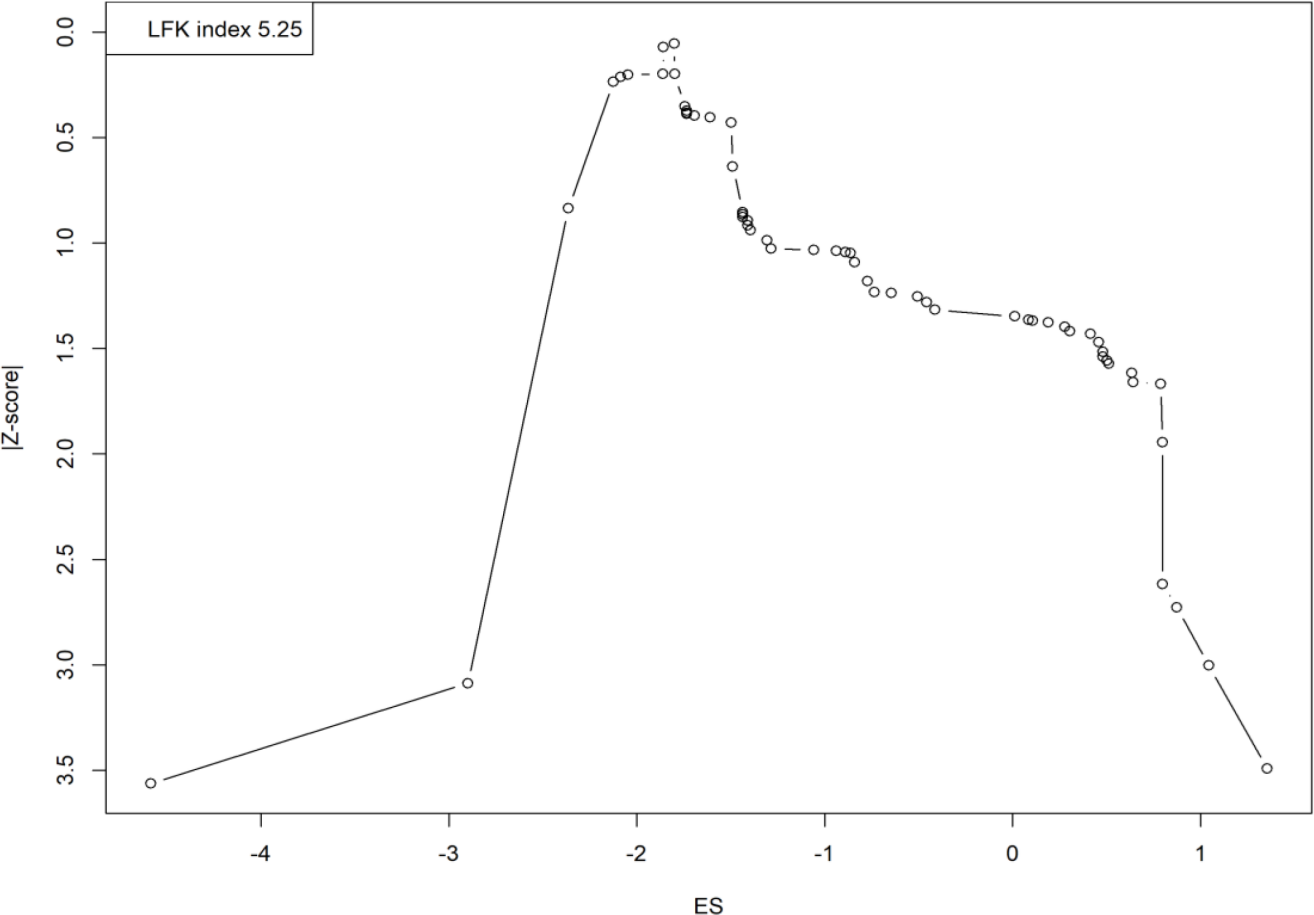
Doi plot illustrating publication bias in studies on fluoroquinolone resistance on UTIs.

The DOI plot showed asymmetry (LFK index: 5.25), indicating publication bias. Smaller studies or selective reporting may have influenced the results, requiring careful interpretation (**Figure 4**).

### Heterogeneity exploration

**Figure 5:**
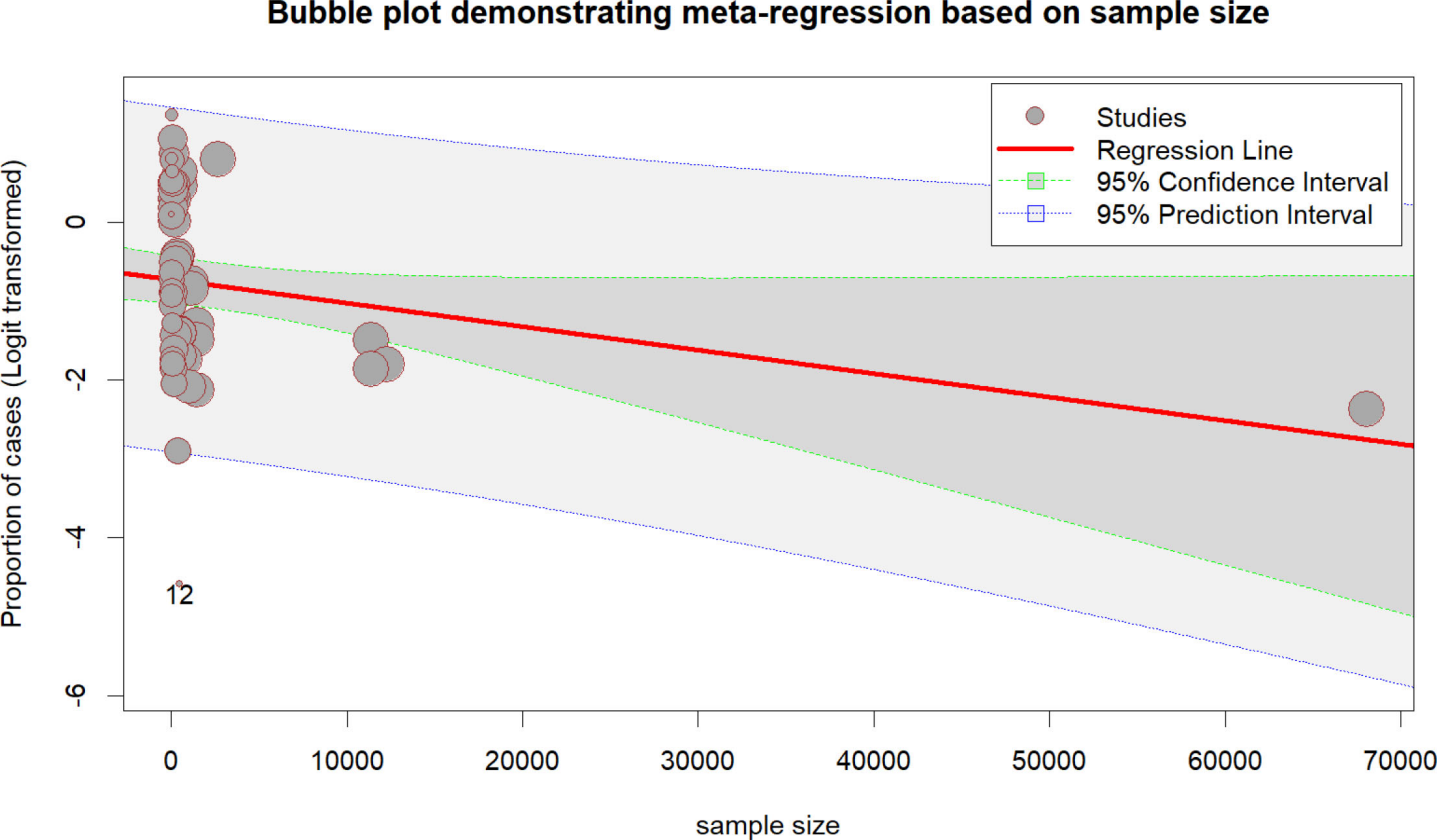
Bubble plot representing meta-regression results.

**Figure 6:**
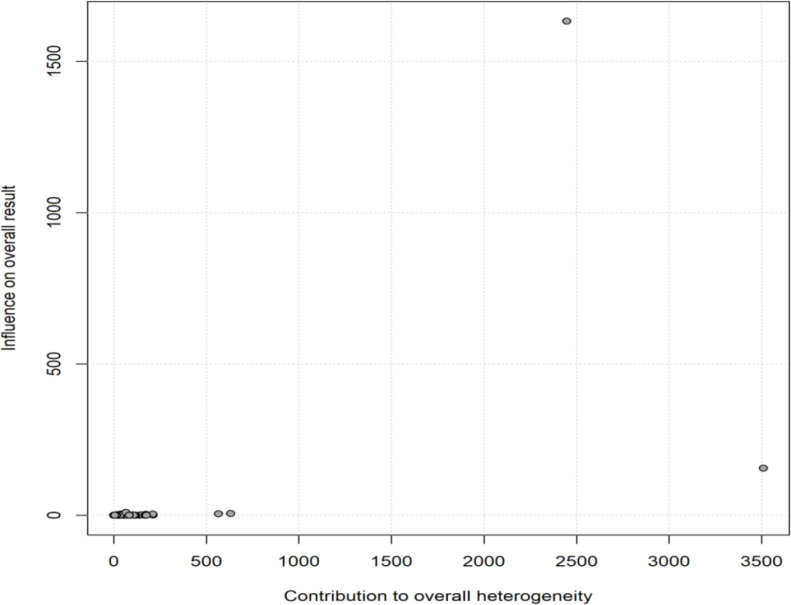
Baujat plot highlighting study heterogeneity.

The meta-regression showed a negative association between sample size and fluoroquinolone resistance, with smaller studies reporting higher rates and larger studies showing lower rates. The p-value of 0.0622 suggests a trend requiring further investigation (**Figure 5**). The Baujat plot showed one study with high heterogeneity and influence on the overall results. Most studies clustered near the origin, indicating minimal impact on heterogeneity or the pooled estimate (**Figure 6**).

### Subgroup analysis by country

Subgroup analysis by country demonstrated substantial geographic variation in fluoroquinolone resistance among *Escherichia coli* isolates causing urinary tract infections (**Table 2**). For ciprofloxacin, resistance prevalence ranged from 13% (95% CI: 13%–14%) in France to 79% (95% CI: 64%–91%) in Spain. High resistance rates were observed in China (63%, 95% CI: 59%–67%), Mexico (62%, 95% CI: 52%–71%), Bangladesh (66%, 95% CI: 46%–82%), and India (50%, 95% CI: 43%–58%). Moderate prevalence was reported in Iran (31%, 95% CI: 26%–37%), Turkey (24%, 95% CI: 22%–27%), and Nepal (32%, 95% CI: 22%–45%), while lower estimates were observed in Romania (15%, 95% CI: 13%–18%) and the United States (18%, 95% CI: 16%–20%) (**Supplementary Figure 1**).

**Table 2:** Subgroup analysis by country.

Country	Ciprofloxacin	Levofloxacin	Norfloxacin
Iran	0.31 (0.26–0.37)	0.15 (0.09–0.23)	0.15 (0.09–0.23)
Saudi Arabia	0.17 (0.11–0.23)	—	—
France	0.13 (0.13–0.14)	0.62 (0.54–0.69)	—
USA	0.18 (0.16–0.20)	0.11 (0.09–0.12)	—
China	0.63 (0.59–0.67)	0.58 (0.54–0.62)	0.28 (0.18–0.41)
Turkey	0.24 (0.22–0.27)	0.01 (0.00–0.02)*	—
Romania	0.15 (0.13–0.18)	0.15 (0.12–0.18)	0.15 (0.12–0.18)
India	0.50 (0.43–0.58)	0.74 (0.65–0.82)	0.74 (0.65–0.82)
Poland	0.40 (0.35–0.45)	—	—
Nepal	0.32 (0.22–0.45)	0.52 (0.40–0.64)	0.52 (0.40–0.64)
Mexico	0.62 (0.52–0.71)	0.61 (0.51–0.71)	0.61 (0.51–0.71)
Spain	0.79 (0.64–0.91)	—	—
Egypt	0.19 (0.15–0.24)	0.19 (0.15–0.24)	0.19 (0.15–0.24)
Bangladesh	0.66 (0.46–0.82)	0.69 (0.49–0.85)	0.69 (0.49–0.85)
Canada	0.18 (0.18–0.19)	—	—
Venezuela	0.29 (0.21–0.39)	—	—
Brazil	0.20 (0.16–0.23)	0.20 (0.16–0.24)	0.20 (0.16–0.24)

*Single study estimate.

Levofloxacin resistance also varied widely across countries (**Supplementary Figure 2**). The highest prevalence was reported in India (74%, 95% CI: 65%–82%), followed by Bangladesh (69%, 95% CI: 49%–85%), France (62%, 95% CI: 54%–69%), Mexico (61%, 95% CI: 51%–71%), and China (58%, 95% CI: 54%–62%). In contrast, lower resistance rates were observed in the United States (11%, 95% CI: 9%–12%), Iran (15%, 95% CI: 9%–23%), and Romania (15%, 95% CI: 12%–18%). An exceptionally low estimate was identified in Turkey (1%, 95% CI: 0%–2%), although this was based on a single study and should be interpreted cautiously.

For norfloxacin, resistance patterns were similarly heterogeneous (**Supplementary Figure 3**). High prevalence was observed in India (74%, 95% CI: 65%–82%), Bangladesh (69%, 95% CI: 49%–85%), and Mexico (61%, 95% CI: 51%–71%). Lower resistance estimates were reported in Iran (15%, 95% CI: 9%–23%), Romania (15%, 95% CI: 12%–18%), and Egypt (19%, 95% CI: 15%–24%), while intermediate levels were observed in China (28%, 95% CI: 18%–41%), Brazil (20%, 95% CI: 16%–24%), and Nepal (52%, 95% CI: 40%–64%).

### Subgroup analysis by study setting

Subgroup analysis by study setting revealed notable differences in fluoroquinolone resistance across community, hospital, and mixed populations (**Table 3**). For ciprofloxacin, resistance prevalence was lowest in community-based studies at 16% (95% CI: 16%–17%), increased in hospital settings to 21% (95% CI: 20%–21%), and was highest in mixed populations at 26% (95% CI: 23%–28%) (**Supplementary Figure 4**).

**Table 3:** Subgroup analysis by setting.

Study setting	Ciprofloxacin	Levofloxacin	Norfloxacin
Community	0.16 (0.16–0.17)	0.24 (0.21–0.27)	0.20 (0.16–0.24)
Hospital	0.21 (0.20–0.21)	0.26 (0.24–0.28)	0.46 (0.42–0.51)
Mixed	0.26 (0.23–0.28)	0.01 (0.00–0.02)*	0.19 (0.15–0.24)

*Single study estimate.

A similar pattern was observed for levofloxacin, with resistance rates of 24% (95% CI: 21%–27%) in community settings and 26% (95% CI: 24%–28%) in hospital-based studies. In contrast, the estimate for mixed settings was markedly lower at 1% (95% CI: 0%–2%), although this result was derived from a single study and should be interpreted with caution (**Supplementary Figure 5**).

For norfloxacin, resistance prevalence was substantially higher in hospital settings at 46% (95% CI: 42%–51%), compared with 20% (95% CI: 16%–24%) in community-based studies and 19% (95% CI: 15%–24%) in mixed populations (**Supplementary Figure 6**). This pronounced difference suggests a higher burden of resistance in healthcare-associated infections.

### Subgroup analysis by study design

Subgroup analysis by study design demonstrated variability in fluoroquinolone resistance estimates across cross-sectional, retrospective, and prospective studies (**Table 4**). For ciprofloxacin, resistance prevalence was 20% (95% CI: 19%–20%) in cross-sectional studies and 17% (95% CI: 16%–18%) in retrospective studies, while higher estimates were observed in prospective studies at 29% (95% CI: 25%–33%) (**Supplementary Figure 7**).

**Table 4:** Subgroup analysis by study design.

Study design	Ciprofloxacin	Levofloxacin	Norfloxacin
Cross-sectional	0.20 (0.19–0.20)	0.17 (0.14–0.20)	0.30 (0.27–0.33)
Retrospective	0.17 (0.16–0.18)	0.21 (0.20–0.23)	0.28 (0.18–0.41)*
Prospective	0.29 (0.25–0.33)	0.62 (0.54–0.69)*	—

*Single study estimate.

For levofloxacin, resistance prevalence was 17% (95% CI: 14%–20%) in cross-sectional studies and 21% (95% CI: 20%–23%) in retrospective studies. A substantially higher estimate was reported in prospective studies at 62% (95% CI: 54%–69%); however, this result was based on a single study and should be interpreted with caution (**Supplementary Figure 8**).

In the case of norfloxacin, resistance prevalence was relatively consistent across study designs, with 30% (95% CI: 27%–33%) reported in cross-sectional studies and 28% (95% CI: 18%–41%) in retrospective studies. No prospective studies were available for norfloxacin (**Supplementary Figure 9**).

## Discussion

This meta-analysis revealed a pooled prevalence of fluoroquinolone resistance in *Escherichia coli* isolates from UTIs at 31.09% (95% CI: 24.89%–38.05%), with ciprofloxacin resistance at 30.32% (95% CI: 22.60%–39.34%) and pefloxacin showing the highest resistance at 68.75% (95% CI: 55.94%–79.76%). Resistance rates varied widely by region, with higher prevalence in countries like China, Iran, and Bangladesh, reflecting differences in antibiotic use, regulation, and healthcare practices. These findings underscore the global challenge of fluoroquinolone resistance and the urgent need for targeted interventions.

Previous studies support these findings, particularly for ciprofloxacin resistance. *Yaping Wu* et al (Wu et al., 2024). reported rates exceeding 50%, significantly higher than the meta-analysis estimate, and noted lower resistance for newer drugs like levofloxacin. *Chang* et al (Chang et al., 2010). linked resistance to gyrA mutations, a mechanism also observed in *E. coli*. Getachew Tadesse et al. highlighted ciprofloxacin resistance driven by genetic mutations and plasmid-mediated mechanisms. Regional variability was noted by *Farzad Khademi* et al (Khademi and Sahebkar, 2020)., attributing it to antibiotic misuse, while *Vishal Goyal* et al (Goyal et al., 2017). emphasized the role of misuse in driving resistance to tuberculosis, a trend paralleling findings in *E. coli*.

Prattanaumpawan et al (Rattanaumpawan et al., 2010). and *van der Starre* et al (Van der Starre et al., 2011). identified critical risk factors for fluoroquinolone resistance. These included recent antibiotic use, hospitalizations, urinary catheter use, and underlying conditions such as recurrent infections or chronic respiratory disease. Both studies emphasized that fluoroquinolone resistance spans hospital and community-acquired infections. These findings align with this meta-analysis, highlighting the importance of addressing resistance risk factors across healthcare and community settings.

A notable finding of this meta-analysis is the extremely high heterogeneity observed across studies (I² ≈ 99%), which likely reflects genuine differences rather than methodological inconsistency alone. Variability in resistance estimates may be attributed to geographic differences in antibiotic prescribing practices, healthcare settings, patient populations, and study designs. Although subgroup analyses by country, study setting, and study design provided some insights into potential sources of heterogeneity, substantial variability remained. Therefore, the pooled estimates should be interpreted cautiously as an overall summary across diverse contexts rather than a uniform global prevalence. The inclusion of prediction intervals further highlights the wide range of resistance rates that may be expected in different settings, underscoring the importance of local surveillance data to guide clinical decision-making and antimicrobial stewardship strategies.

The extreme heterogeneity observed is a defining feature of global antimicrobial resistance (AMR) data. This degree of variability suggests that fluoroquinolone resistance is driven by highly localized ecological “niches,” shaped by factors such as regional over-the-counter (OTC) antibiotic regulations, hospital sanitation practices, and environmental runoff.

Consequently, while the pooled estimate of 31.09% provides a summary of the available global literature, its interpretability remains limited in the absence of granular, longitudinal data. In this context, the broad prediction intervals reported in this study may be more clinically informative than the pooled mean, as they better capture the real-world variability. Specifically, they reflect the high likelihood of encountering near-universal resistance in high-burden settings, contrasted with relatively preserved antibiotic efficacy in lower-burden regions.

The observed pooled prevalence of 31.09% underscores the evolutionary success of *Escherichia coli* under intense selective pressure. This resistance is rarely the result of isolated events but rather reflects a complex interplay between chromosomal mutations and plasmid-mediated quinolone resistance (PMQR) (Redgrave et al., 2014; Yang et al., 2025). While mutations in the quinolone resistance–determining regions (QRDRs) of the *gyrA* and *parC* genes remain the primary drivers of high-level resistance, the role of PMQR genes such as *qnr* variants, *aac(6’)-Ib-cr*, and efflux pump regulators cannot be overstated. These plasmid-borne elements often confer low-level resistance that acts as an “evolutionary springboard,” enabling bacterial populations to survive subtherapeutic antibiotic concentrations and subsequently acquire the chromosomal mutations required for clinical failure (Zheng et al., 2025).

Furthermore, the phenomenon of co-selection complicates this landscape. Fluoroquinolone resistance genes are frequently co-located on large multidrug-resistant (MDR) plasmids alongside β-lactamases (e.g., CTX-M) (Cantón and Coque, 2006). Consequently, the use of cephalosporins in hospital settings may inadvertently sustain fluoroquinolone resistance even in the absence of direct quinolone exposure. This genetic linkage likely contributes to the persistently higher resistance rates observed in hospital settings (e.g., 46% for norfloxacin) compared with community isolates (Hooper and Jacoby, 2015).

When contextualized within the existing literature, a clear temporal and geographic divergence emerges. Our estimate of 30.32% for ciprofloxacin resistance aligns with, and in some regions exceeds, trends reported by the World Health Organization through the Global Antimicrobial Resistance Surveillance System (World Health Organization, 2022). While studies from the early 2010s often reported resistance rates below 20% in many regions, our findings suggest that this “resistance floor” has shifted upward over time. Notably, our results indicate a higher burden than that reported by Wu et al., who observed lower resistance rates for newer-generation agents such as levofloxacin. This discrepancy may be explained by the “mutant prevention concentration” (MPC) theory, whereby older fluoroquinolones with less optimized pharmacokinetic/pharmacodynamic (PK/PD) profiles may facilitate more rapid selection of resistant strains (Drlica, 2003; Zhuang et al., 2023).

Geographically, the marked contrast between relatively low resistance in North America (11%–18%) and substantially higher rates in countries such as China and India (>60%) reflects more than differences in prescribing practices. These patterns likely indicate broader systemic challenges consistent with a “One Health” framework, including environmental contamination from pharmaceutical manufacturing, unregulated over-the-counter antibiotic access, and inadequate antimicrobial stewardship (Larsson, 2014; Li et al., 2025). Together, these factors contribute to the establishment of persistent reservoirs of resistant organisms. Consequently, global pooled estimates may be less informative for clinical decision-making than context-specific data, and the wide prediction intervals observed in this analysis highlight the potential for near-universal resistance in high-burden regions.

This meta-analysis highlights critical implications for clinical practice, public health policy, and future research. In practice, fluoroquinolones should not be used indiscriminately, especially in regions with high resistance rates. Clinicians should base treatment decisions on local resistance patterns and use alternative antibiotics guided by culture and susceptibility testing. Rapid molecular diagnostic tools should be implemented to enable targeted therapy, reducing reliance on empirical fluoroquinolone use. For policymakers, strengthening antimicrobial stewardship programs is essential to curb inappropriate antibiotic use. Public awareness campaigns should educate communities on the dangers of misuse and resistance. Regulations must enforce prescription-only access and limit over-the-counter antibiotic sales. Investment in surveillance systems is critical to monitor resistance patterns and guide policies. Efforts should also support the development of new antimicrobial agents and alternative treatments to address emerging resistance challenges.

It is critical to note that the global pooled resistance prevalence of 31.09% serves as a broad epidemiological benchmark rather than a direct clinical guide. Due to the extreme geographic and setting-based variability, this estimate has limited direct applicability to individual bedside decision-making. For instance, empirical ciprofloxacin use may remain viable in regions reporting <15% resistance (e.g., parts of North America), while it is likely to result in clinical failure in regions exceeding 60% (e.g., China or Spain). Clinicians must prioritize local antibiograms and institutional surveillance over global averages. The primary clinical utility of this meta-analysis is to highlight the systemic decline of fluoroquinolone reliability globally and to reinforce the urgent need for rapid, point-of-care susceptibility testing to replace empirical ‘one-size-fits-all’ prescribing.

This meta-analysis has several strengths. It synthesized data from 36 studies across diverse regions, offering a global perspective on fluoroquinolone resistance in *E. coli* UTIs. The inclusion of cross-sectional, retrospective, and prospective studies provided robust insights into resistance patterns across various settings. Subgroup analyses of multiple fluoroquinolones allowed for detailed drug-specific comparisons. Advanced statistical methods, including random-effects modeling and sensitivity analysis, accounted for heterogeneity and identified influential studies.

Although most included studies were of moderate quality, variability in study design, case definitions, and outcome assessment may have contributed to heterogeneity in the pooled estimates. Differences in how UTIs were defined and how resistance was measured could influence reported prevalence rates, highlighting the importance of standardized methodologies in future research. Although subgroup or sensitivity analyses based on study quality were considered, they were not feasible due to the limited number of studies within each quality category and the relatively narrow range of quality scores. As most studies were of moderate to high quality, meaningful stratified comparisons could not be performed. This limitation may affect the ability to fully assess the impact of study quality on the pooled estimates and should be considered when interpreting the results.

Despite its strengths, this review has limitations. Most included studies were observational, introducing risks of selection bias and confounding. Regional disparities were evident, with the underrepresentation of low-income areas. Reliance on published studies likely introduced publication bias, and inconsistent resistance definitions reduced comparability. Excluding non-English studies and older data may have further limited the scope, while variability in study quality impacted the overall reliability of results. The extreme heterogeneity observed remains a significant limitation, indicating that fluoroquinolone resistance is driven by highly localized clinical and regulatory factors. Consequently, the global pooled estimate acts as a macro-level indicator of selective pressure rather than a uniform clinical reality. Due to data gaps in the primary literature, we could not perform granular stratifications by time trends or sub-regional burdens. This prevents a definitive assessment of how resistance has evolved over the 15-year study period. While the use of prediction intervals helps illustrate the wide range of possible resistance levels, these findings should be interpreted with caution and should not supersede local surveillance data when guiding empirical therapy.

Future research should address gaps identified in this review. Standardized definitions and consistent reporting practices are necessary for better comparability across studies. Research should prioritize underrepresented regions, particularly low-income areas, to provide a comprehensive view of global resistance trends. Molecular studies are needed to explore mechanisms driving resistance and to develop innovative countermeasures. Longitudinal studies should evaluate the impact of stewardship programs and monitor resistance trends over time. Additionally, the role of environmental and agricultural antibiotic use in driving resistance requires further investigation to inform broader policy actions.

## Conclusion

This meta-analysis demonstrates a substantial prevalence of fluoroquinolone resistance in Escherichia coli isolates from urinary tract infections, with marked regional variation particularly in countries such as China, Iran, and Bangladesh. Among the evaluated agents, ciprofloxacin showed considerable resistance, while pefloxacin exhibited the highest resistance rates. These findings underscore the urgent need for strengthened antibiotic stewardship programs, stricter regulatory oversight, and more robust surveillance systems. Future research should focus on standardizing resistance definitions, elucidating the genetic mechanisms underlying resistance, and addressing data gaps from underrepresented regions to better inform targeted interventions and monitor evolving resistance trends. Although this meta-analysis provides a comprehensive global overview, the high degree of heterogeneity highlights that antimicrobial resistance is fundamentally a localized phenomenon. Consequently, global estimates should not be used in isolation to guide empirical therapy. Rather, they should serve as a catalyst for strengthening local surveillance systems and advancing toward precision antimicrobial stewardship tailored to regional resistance patterns.

## Data Availability

The original contributions presented in the study are included in the article/**Supplementary Material**. Further inquiries can be directed to the corresponding authors.

## References

[B1] Afsharikhah S. Ghanbarpour R. Mohseni P. Adib N. Bagheri M. Jajarmi M. (2023). High prevalence of β-lactam and fluoroquinolone resistance in various phylotypes of Escherichia coli isolates from urinary tract infections in Jiroft city, Iran. BMC Microbiol. 23, 114. doi:10.1186/s12866-023-02860-7. PMID: 37087426 PMC10122366

[B2] Ahmed S. K. Hussein S. Qurbani K. Ibrahim R. H. Fareeq A. Mahmood K. A. . (2024). Antimicrobial resistance: impacts, challenges, and future prospects. J. Medicine Surgery Public Health 2, 100081. doi:10.1016/j.glmedi.2024.100081. PMID: 38826717

[B3] Akgoz M. Akman I. Ates A. B. Celik C. Keskin B. Ozmen Capin B. B. . (2020). Plasmidic fluoroquinolone resistance genes in fluoroquinolone-resistant and/or extended spectrum beta-lactamase-producing Escherichia coli strains isolated from pediatric and adult patients diagnosed with urinary tract infection. Microb. Drug Resist. 26, 1334–1341. doi:10.1089/mdr.2020.0007. PMID: 32407158

[B4] Asadi S. Kargar M. Solhjoo K. Najafi A. Ghorbani-Dalini S. (2014). The association of virulence determinants of uropathogenic Escherichia coli with antibiotic resistance. Jundishapur J. Microbiol. 7. doi:10.5812/jjm.9936. PMID: 25147722 PMC4138644

[B5] Ashraf M. S. Gaur S. Bushen O. Y. Chopra T. Chung P. Clifford K. . (2020). Diagnosis, treatment, and prevention of urinary tract infections in post-acute and long-term care settings: a consensus statement from AMDA's Infection Advisory Subcommittee. J. Am. Med. Directors Assoc. 21, 12–24, e2. doi:10.1016/j.jamda.2019.11.004. PMID: 31888862

[B6] Baimakhanova B. Sadanov A. Trenozhnikova L. Balgimbaeva A. Baimakhanova G. Orasymbet S. . (2025). Understanding the burden and management of urinary tract infections in women. Diseases 13, 59. doi:10.3390/diseases13020059. PMID: 39997066 PMC11854091

[B7] Bazaid A. S. Saeed A. Alrashidi A. Alrashidi A. Alshaghdali K. A Hammam S. . (2021). Antimicrobial surveillance for bacterial uropathogens in Ha’il, Saudi Arabia: A five-year multicenter retrospective study. Infection Drug Resistance, 1455–1465. doi:10.2147/idr.s299846. PMID: 33888997 PMC8057796

[B8] Bhatt S. Chatterjee S. (2022). Fluoroquinolone antibiotics: occurrence, mode of action, resistance, environmental detection, and remediation–a comprehensive review. Environ. pollut. 315, 120440. doi:10.1016/j.envpol.2022.120440. PMID: 36265724

[B9] Biguenet A. Bouxom H. Bertrand X. Slekovec C. (2023). Antibiotic resistance in elderly patients: comparison of Enterobacterales causing urinary tract infections between community, nursing homes and hospital settings. Infect. Dis. Now 53, 104640. doi:10.1016/j.idnow.2022.12.005. PMID: 36621613

[B10] Brosh-Nissimov T. Navon-Venezia S. Keller N. Amit S. (2019). Risk analysis of antimicrobial resistance in outpatient urinary tract infections of young healthy adults. J. Antimicrob. Chemother. 74, 499–502. doi:10.1093/jac/dky424. PMID: 30357329

[B11] Can F. Azap O. K. Seref C. Ispir P. Arslan H. Ergonul O. (2015). Emerging Escherichia coli O25b/ST131 clone predicts treatment failure in urinary tract infections. Clin. Infect. Dis. 60, 523–527. doi:10.1093/cid/ciu864. PMID: 25378460

[B12] Cantón R. Coque T. M. (2006). The CTX-M β-lactamase pandemic. Curr. Opin. Microbiol. 9, 466–475. 16942899 10.1016/j.mib.2006.08.011

[B13] Chang K. C. Yew W. W. Chan R. C. Y. (2010). Rapid assays for fluoroquinolone resistance in Mycobacterium tuberculosis: a systematic review and meta-analysis. J. Antimicrob. Chemother. 65, 1551–1561. doi:10.1093/jac/dkq202. PMID: 20542907

[B14] Cristea V. C. Gheorghe I. Czobor Barbu I. Popa L. I. Ispas B. Grigore G. A. . (2019). Snapshot of phylogenetic groups, virulence, and resistance markers in Escherichia coli uropathogenic strains isolated from outpatients with urinary tract infections in Bucharest, Romania. BioMed. Res. Int. 2019, 5712371. doi:10.1155/2019/5712371. PMID: 31236408 PMC6545812

[B15] Damlin A. (2020). “ Responsible Antibiotic Use And Diagnostic Challenges In Infectious Diseases: Studies in a resource-limited setting and a high-income setting,” in Karolinska institutet (Sweden). Damlin A. RESPONSIBLE ANTIBIOTIC USE AND DIAGNOSTIC CHALLENGES IN INFECTIOUS DISEASES: Studies in a resource-limited setting and a high-income setting: Karolinska Institutet (Sweden); 2020.

[B16] Demir M. Kazanasmaz H. (2020). Uropathogens and antibiotic resistance in the community and hospital-induced urinary tract infected children. J. Glob. Antimicrob. Resist. 20, 68–73. doi:10.1016/j.jgar.2019.07.019. PMID: 31340182

[B17] Demirci M. Ünlü Ö. Tosun A.İ. (2019). Detection of O25b-ST131 clone, CTX-M-1 and CTX-M-15 genes via real-time PCR in Escherichia coli strains in patients with UTIs obtained from a university hospital in Istanbul. J. Infection Public Health 12, 640–644. doi:10.1016/j.jiph.2019.02.017. PMID: 30826300

[B18] de Souza da-Silva A. P. de Sousa V. S. de Araújo Longo L. G. Caldera S. Baltazar I. C. L. Bonelli R. R. . (2020). Prevalence of fluoroquinolone-resistant and broad-spectrum cephalosporin-resistant community-acquired urinary tract infections in Rio de Janeiro: impact of Escherichia coli genotypes ST69 and ST131. Infection Genet. Evol. 85, 104452. doi:10.1016/j.meegid.2020.104452. PMID: 32634601

[B19] Drlica K. (2003). The mutant selection window and antimicrobial resistance. J. Antimicrob. Chemother. 52, 11–17. doi:10.1093/jac/dkg269. PMID: 12805267

[B20] Etienne M. Lefebvre E. Frebourg N. Hamel H. Pestel-Caron M. Caron F. . (2014). Antibiotic treatment of acute uncomplicated cystitis based on rapid urine test and local epidemiology: lessons from a primary care series. BMC Infect. Dis. 14, 1–8. doi:10.1186/1471-2334-14-137. PMID: 24612927 PMC3975248

[B21] Faine B. A. Rech M. A. Vakkalanka P. Gross A. Brown C. Harding S. J. . (2022). High prevalence of fluoroquinolone‐resistant UTI among US emergency department patients diagnosed with urinary tract infection, 2018–2020. Acad. Emergency Med. 29, 1096–1105. doi:10.1093/ofid/ofab466.1611 PMC9543902 35652493

[B22] Findlay J. Gould V. C. North P. Bowker K. E. Williams M. O. MacGowan A. P. . (2020). Characterization of cefotaxime-resistant urinary Escherichia coli from primary care in South-West England 2017–18. J. Antimicrob. Chemother. 75, 65–71. doi:10.1093/jac/dkz397. PMID: 31538190

[B23] Fleming V. H. White B. P. Southwood R. (2014). Resistance of Escherichia coli urinary isolates in ED-treated patients from a community hospital. Am. J. Emergency Med. 32, 864–870. doi:10.1016/j.ajem.2014.04.033. PMID: 24877721

[B24] Fromer D. L. Cheng W. Y. Gao C. Mahendran M. Hilts A. Duh M. S. . (2024). Likelihood of antimicrobial resistance in urinary E. coli isolates among US female patients with recurrent vs non-recurrent uncomplicated urinary tract infection. Urology. doi:10.1016/j.urology.2024.02.047. PMID: 38467284

[B25] Govindarajan D. K. Kandaswamy K. (2022). Virulence factors of uropathogens and their role in host pathogen interactions. Cell Surf. 8, 100075. doi:10.1016/j.tcsw.2022.100075. PMID: 35198842 PMC8841375

[B26] Goyal V. Kadam V. Narang P. Singh V. (2017). Prevalence of drug-resistant pulmonary tuberculosis in India: systematic review and meta-analysis. BMC Public Health 17, 1–21. doi:10.1186/s12889-017-4779-5. PMID: 29041901 PMC5645895

[B27] Guzmán M. Salazar E. Cordero V. Castro A. Villanueva A. Rodulfo H. . (2019). Multidrug resistance and risk factors associated with community-acquired urinary tract infections caused by Escherichia coli in Venezuela. Biomédica 39, 96–106. 31529852 10.7705/biomedica.v39i2.4030

[B28] Harwalkar A. Gupta S. Rao A. Srinivasa H. (2014). Lower prevalence of hlyD, papC and cnf-1 genes in ciprofloxacin-resistant uropathogenic Escherichia coli than their susceptible counterparts isolated from southern India. J. Infection Public Health 7, 413–419. doi:10.1016/j.jiph.2014.04.002. PMID: 24861644

[B29] Hooper D. C. Jacoby G. A. (2015). Mechanisms of drug resistance: quinolone resistance. Ann. N. Y. Acad. Sci. 1354, 12–31. doi:10.1111/nyas.12830. PMID: 26190223 PMC4626314

[B30] Islam M. A. Islam M. R. Khan R. Amin M. B. Rahman M. Hossain M. I. . (2022). Prevalence, etiology and antibiotic resistance patterns of community-acquired urinary tract infections in Dhaka, Bangladesh. PloS One 17, e0274423. doi:10.1371/journal.pone.0274423. PMID: 36107878 PMC9477272

[B31] Jurałowicz E. Bartoszko-Tyczkowska A. Tyczkowska-Sieroń E. Kurnatowska I. (2020). Etiology and bacterial susceptibility to antibiotics in patients with recurrent lower urinary tract infections. Polskie Archiwum Medycyny Wewnętrznej 130. 10.20452/pamw.15284 32250578

[B32] Kamei J. Fujimura T. (2023). Urinary tract infection in patients with lower urinary tract dysfunction. J. Infect. Chemother. 29, 744–748. doi:10.1016/j.jiac.2023.04.019. PMID: 37149001

[B33] Khademi F. Sahebkar A. (2020). Prevalence of fluoroquinolone‐resistant Campylobacter species in Iran: a systematic review and meta‐analysis. Int. J. Microbiol. 2020, 8868197. doi:10.1155/2020/8868197. PMID: 33488728 PMC7803110

[B34] Kotb D. N. Mahdy W. K. Mahmoud M. S. Khairy R. M. (2019). Impact of co-existence of PMQR genes and QRDR mutations on fluoroquinolones resistance in Enterobacteriaceae strains isolated from community and hospital acquired UTIs. BMC Infect. Dis. 19, 1–8. doi:10.1186/s12879-019-4606-y. PMID: 31752702 PMC6868749

[B35] Kresken M. Pfeifer Y. Hafner D. Wresch R. Körber-Irrgang B. Chemotherapy WPARotP-E-Sf (2014). Occurrence of multidrug resistance to oral antibiotics among Escherichia coli urine isolates from outpatient departments in Germany: extended-spectrum β-lactamases and the role of fosfomycin. Int. J. Antimicrob. Agents 44, 295–300. doi:10.1016/j.ijantimicag.2014.05.020. PMID: 25223936

[B36] Kushwaha A. Pokharel K. Kadel A. R. (2021). Antibiotic resistance to Escherichia coli among urine culture-positive patients in a tertiary care hospital in Nepal: A descriptive cross-sectional study. JNMA: J. Nepal Med. Assoc. 59, 39. doi:10.31729/jnma.5545. PMID: 34508456 PMC7893388

[B37] Larsson D. J. (2014). Antibiotics in the environment. Upsala J. Med. Sci. 119, 108–112. doi:10.3109/03009734.2014.896438. PMID: 24646081 PMC4034546

[B38] Leforestier A. Vibet M.-A. Gentet N. Javaudin F. Le Bastard Q. Montassier E. . (2020). Modeling the risk of fluoroquinolone resistance in non-severe community-onset pyelonephritis. Eur. J. Clin. Microbiol. Infect. Dis. 39, 1123–1127. doi:10.1007/s10096-020-03830-x. PMID: 31997098

[B39] Li J. Jiang F. Xie A. Jiang Y. (2022). Analysis of the distribution and drug resistance of pathogens in patients with urinary tract infection in the Eastern Chongming area of Shanghai from 2018 to 2020. Infection Drug Resistance, 6413–6422. doi:10.2147/idr.s384515. PMID: 36345539 PMC9636864

[B40] Li Y.-K. Li W.-R. Ren H. Xiao C.-L. Guo Z. Luo J.-Q. (2025). Gut microbiome-targeted therapeutics for chronic kidney disease: Comparative efficacy of probiotic and microbial preparations. Inflammopharmacology, 1–17. doi:10.1007/s10787-025-02044-x. PMID: 41236711

[B41] López-Banda D. A. Carrillo-Casas E. M. Leyva-Leyva M. Orozco-Hoyuela G. Manjarrez-Hernández Á.H. Arroyo-Escalante S. . (2014). Identification of virulence factors genes in Escherichia coli isolates from women with urinary tract infection in Mexico. BioMed. Res. Int. 2014, 959206. doi:10.1155/2014/959206. PMID: 24895634 PMC4026957

[B42] Loras C. Mendes A. C. Peixe L. Novais Â. Alós J.-I. (2020). Escherichia coli resistant to fosfomycin from urinary tract infections: detection of the fosA3 gene in Spain. J. Global Antimicrob. Resist. 21, 414–416. doi:10.1016/j.jgar.2020.01.023. PMID: 32061811

[B43] Maillard J.-Y. Bloomfield S. F. Courvalin P. Essack S. Y. Gandra S. Gerba C. P. . (2020). Reducing antibiotic prescribing and addressing the global problem of antibiotic resistance by targeted hygiene in the home and everyday life settings: a position paper. Am. J. Infection Control 48, 1090–1099. doi:10.1016/j.ajic.2020.04.011. PMID: 32311380 PMC7165117

[B44] Meng Z. Wang J. Lin L. Wu C. (2024). Sensitivity analysis with iterative outlier detection for systematic reviews and meta‐analyses. Stat. Med. 43, 1549–1563. doi:10.1002/sim.10008. PMID: 38318993 PMC10947935

[B45] Mheissen S. Khan H. Normando D. Vaiid N. Flores-Mir C. (2024). Do statistical heterogeneity methods impact the results of meta-analyses? A meta epidemiological study. PloS One 19, e0298526. doi:10.1371/journal.pone.0298526. PMID: 38502662 PMC10950254

[B46] Milano A. Sulejmani A. Intra J. Sala M. R. Leoni V. Carcione D. (2022). Antimicrobial resistance trends of Escherichia coli isolates from outpatient and inpatient urinary infections over a 20-year period. Microb. Drug Resist. 28, 63–72. doi:10.1089/mdr.2021.0010. PMID: 34520265

[B47] Muteeb G. Rehman M. T. Shahwan M. Aatif M. (2023). Origin of antibiotics and antibiotic resistance, and their impacts on drug development: a narrative review. Pharmaceuticals 16, 1615. doi:10.3390/ph16111615. PMID: 38004480 PMC10675245

[B48] Naber K. G. Wagenlehner F. Kresken M. Cheng W. Y. Catillon M. Duh M. S. . (2023). Escherichia coli resistance, treatment patterns and clinical outcomes among females with uUTI in Germany: a retrospective physician-based chart review study. Sci. Rep. 13, 12077. doi:10.1038/s41598-023-38919-8. PMID: 37495602 PMC10372039

[B49] Nasrollahian S. Graham J. P. Halaji M. (2024). A review of the mechanisms that confer antibiotic resistance in pathotypes of E. coli. Front. Cell. Infect. Microbiol. 14, 1387497. doi:10.3389/fcimb.2024.1387497. PMID: 38638826 PMC11024256

[B50] Niranjan V. Malini A. (2014). Antimicrobial resistance pattern in Escherichia coli causing urinary tract infection among inpatients. Indian J. Med. Res. 139, 945–948. doi:10.5005/jp/books/11823_8 25109731 PMC4165009

[B51] Rahman S. R. Ahmed M. F. Begum A. (2014). Occurrence of urinary tract infection in adolescent and adult women of shanty town in Dhaka City, Bangladesh. Ethiopian J. Health Sci. 24, 145–152. doi:10.4314/ejhs.v24i2.7. PMID: 24795516 PMC4006209

[B52] Rattanaumpawan P. Tolomeo P. Bilker W. Fishman N. Lautenbach E. (2010). Risk factors for fluoroquinolone resistance in Gram-negative bacilli causing healthcare-acquired urinary tract infections. J. Hosp. Infection 76, 324–327. doi:10.1016/j.jhin.2010.05.023. PMID: 20643497 PMC2962678

[B53] Redgrave L. S. Sutton S. B. Webber M. A. Piddock L. J. (2014). Fluoroquinolone resistance: mechanisms, impact on bacteria, and role in evolutionary success. Trends Microbiol. 22, 438–445. doi:10.1016/j.tim.2014.04.007. PMID: 24842194

[B54] Rodrigues W. F. Miguel C. B. Nogueira A. P. O. Ueira-Vieira C. Paulino T. D. P. Soares S. D. C. . (2016). Antibiotic resistance of bacteria involved in urinary infections in Brazil: a cross-sectional and retrospective study. Int. J. Environ. Res. Public Health 13, 918. doi:10.3390/ijerph13090918. PMID: 27649224 PMC5036751

[B55] Schwarzer G. (2022). “ Meta‐analysis in R,” in Systematic reviews in health research: meta‐Analysis in context, 510–534.

[B56] Sedighi I. Arabestani M. R. Rahimbakhsh A. Karimitabar Z. Alikhani M. Y. (2015). Dissemination of extended-spectrum β-lactamases and quinolone resistance genes among clinical isolates of uropathogenic Escherichia coli in children. Jundishapur J. Microbiol. 8. doi:10.5812/jjm.19184v2. PMID: 26421128 PMC4584072

[B57] Shafrin J. Marijam A. Joshi A. V. Mitrani-Gold F. S. Everson K. Tuly R. . (2022). Impact of suboptimal or inappropriate treatment on healthcare resource use and cost among patients with uncomplicated urinary tract infection: an analysis of integrated delivery network electronic health records. Antimicrobial Resistance Infection Control 11, 133. doi:10.1186/s13756-022-01170-3. PMID: 36333740 PMC9636777

[B58] Shamim M. A. (2023). Real-life implications of prevalence meta-analyses? Doi plots and prediction intervals are the answer. Lancet Microbe 4, e490. doi:10.1016/s2666-5247(23)00096-4. PMID: 37116520

[B59] Soucy J.-P. Schmidt A. M. Quach C. Buckeridge D. L. (2020). Fluoroquinolone use and seasonal patterns of ciprofloxacin resistance in community-acquired urinary Escherichia coli infection in a large urban center. Am. J. Epidemiol. 189, 215–223. doi:10.1093/aje/kwz239. PMID: 31665215 PMC7217283

[B60] Themphachana M. Kanobthammakul S. Nakaguchi Y. Singkhamanan K. Yadrak P. Sukhumungoon P. (2014). Virulence characteristics and antimicrobial susceptibility of uropathogens from patients on Phuket Island, Thailand. Southeast. Asian J. Trop. Med. Public Health 45, 1090. 25417510

[B61] Timm M. R. Russell S. K. Hultgren S. J. (2024). Urinary tract infections: pathogenesis, host susceptibility and emerging therapeutics. Nat. Rev. Microbiol., 1–15. doi:10.1038/s41579-024-01092-4. PMID: 39251839 PMC13194463

[B62] Urban-Chmiel R. Marek A. Stępień-Pyśniak D. Wieczorek K. Dec M. Nowaczek A. . (2022). Antibiotic resistance in bacteria—a review. Antibiotics 11, 1079. doi:10.3390/antibiotics11081079. PMID: 36009947 PMC9404765

[B63] Van der Starre W. E. Van Nieuwkoop C. Paltansing S. Van't Wout J. W. Groeneveld G. H. Becker M. J. . (2011). Risk factors for fluoroquinolone-resistant Escherichia coli in adults with community-onset febrile urinary tract infection. J. Antimicrob. Chemother. 66, 650–656. doi:10.1093/jac/dkq465. PMID: 21123286

[B64] Wagenlehner F. M. Bjerklund Johansen T. E. Cai T. Koves B. Kranz J. Pilatz A. . (2020). Epidemiology, definition and treatment of complicated urinary tract infections. Nat. Rev. Urol. 17, 586–600. doi:10.1038/s41585-020-0362-4. PMID: 32843751

[B65] Wang L. Qiu H. Tan X. Liu J. Yang T. Jiang C. (2025). Impact of roxadustat on anemia management in infected patients undergoing long-term dialysis: a retrospective cohort analysis. Front. Pharmacol. 16, 1695376. doi:10.3389/fphar.2025.1695376. PMID: 41132544 PMC12540434

[B66] Wang Q. Zhao K. Guo C. Li H. Huang T. Ji J. . (2021). Antibiotic resistance and virulence genes of Escherichia coli isolated from patients with urinary tract infections after kidney transplantation from deceased donors. Infection Drug Resistance, 4039–4046. doi:10.2147/idr.s332897. PMID: 34616161 PMC8487860

[B67] Wang Y. Zhao S. Han L. Guo X. Chen M. Ni Y. . (2014). Drug resistance and virulence of uropathogenic Escherichia coli from Shanghai, China. J. Antibiot. 67, 799–805. doi:10.1038/ja.2014.72. PMID: 24984795

[B68] World Health Organization (2022). “ Global antimicrobial resistance and use surveillance system (GLASS) report 2022,” in World health organization.

[B69] Wu Y. Majidzadeh N. Li Y. Shakourzadeh M. Z. Hajilari S. Kouhsari E. . (2024). Trends of fluoroquinolones resistance in Mycoplasma and Ureaplasma urogenital isolates: systematic review and meta-analysis. J. Global Antimicrob. Resist. 36, 13–25. doi:10.1016/j.jgar.2023.11.007. PMID: 38016593

[B70] Yang Y. Li C. Fan X. Long W. Hu Y. Wang Y. . (2025). Effectiveness of omadacycline in a patient with Chlamydia psittaci and KPC-producing gram-negative bacteria infection. Infection Drug Resistance, 903–908. doi:10.2147/idr.s505311. PMID: 39990782 PMC11844266

[B71] Zhai C. Guyatt G. (2024). “ Fixed-effect and random-effects models in meta-analysis,” in LWW, 1–4. 10.1097/CM9.0000000000002814 PMC10766278 37612263

[B72] Zhao Y.-L. Qu Q. Wang Y.-M. Zhang Y.-T. Qu J. (2026). A disproportionality analysis of adverse events associated with omadacycline based on the FDA adverse event reporting system database. J. Antimicrob. Chemother. 81, dkag006. doi:10.1093/jac/dkag006. PMID: 41578739

[B73] Zheng J. She H. Han R. Tang J. Dou Y. Lu C. . (2025). Dapk2 dysfunction leads to Mic60 lactylation and mitochondrial metabolic reprogramming, promoting lung cancer EGFR-TKI resistance and metastasis. Dev. Cell 60, 3267–3284. doi:10.1016/j.devcel.2025.07.014. PMID: 40812308

[B74] Zhou Y. Zhou Z. Zheng L. Gong Z. Li Y. Jin Y. . (2023). Urinary tract infections caused by uropathogenic Escherichia coli: mechanisms of infection and treatment options. Int. J. Mol. Sci. 24, 10537. doi:10.3390/ijms241310537. PMID: 37445714 PMC10341809

[B75] Zhuang H.-H. Chen Y. Hu Q. Long W.-M. Wu X.-L. Wang Q. . (2023). Efficacy and mortality of ceftazidime/avibactam-based regimens in carbapenem-resistant Gram-negative bacteria infections: a retrospective multicenter observational study. J. Infection Public Health 16, 938–947. doi:10.1016/j.jiph.2023.04.014. PMID: 37087853

